# Regulating Exciton–Phonon Coupling to Achieve a Near‐Unity Photoluminescence Quantum Yield in One‐Dimensional Hybrid Metal Halides

**DOI:** 10.1002/advs.202100786

**Published:** 2021-05-22

**Authors:** Hui Luo, Songhao Guo, Yubo Zhang, Kejun Bu, Haoran Lin, Yingqi Wang, Yanfeng Yin, Dongzhou Zhang, Shengye Jin, Wenqing Zhang, Wenge Yang, Biwu Ma, Xujie Lü

**Affiliations:** ^1^ Center for High Pressure Science and Technology Advanced Research (HPSTAR) 1690 Cailun Rd, Pudong Shanghai 201203 China; ^2^ Department of Physics and Shenzhen Institute for Quantum Science and Engineering Southern University of Science and Technology Shenzhen Guangdong 518055 China; ^3^ Hoffmann Institute of Advanced Materials Shenzhen Polytechnic Shenzhen Guangdong 518055 China; ^4^ State Key Laboratory of Molecular Reaction Dynamics and Dynamics Research Center for Energy and Environmental Materials Dalian Institute of Chemical Physics Chinese Academy of Sciences Dalian Liaoning 116023 China; ^5^ Hawaii Institute of Geophysics and Planetology University of Hawaii Manoa Honolulu HI 96822 USA; ^6^ Department of Chemistry and Biochemistry Florida State University Tallahassee FL 32306 USA

**Keywords:** 1D hybrid metal halides, exciton–phonon coupling, Huang–Rhys factor, pressure regulation, self‐trapped excitons

## Abstract

Low‐dimensional hybrid metal halides are emerging as a highly promising class of single‐component white‐emitting materials for their unique broadband emission from self‐trapped excitons (STEs). Despite substantial progress in the development of these metal halides, many challenges remain to be addressed to obtain a better fundamental understanding of the structure–property relationship and realize the full potentials of this class of materials. Here, via pressure regulation, a near 100% photoluminescence quantum yield (PLQY) of broadband emission is achieved in a corrugated 1D hybrid metal halide C_5_N_2_H_16_Pb_2_Br_6_, which possesses a highly distorted structure with an initial PLQY of 10%. Compression reduces the overlap between STE states and ground state, leading to a suppressed phonon‐assisted non‐radiative decay. The PL evolution is systematically demonstrated to be controlled by the pressure‐regulated exciton–phonon coupling which can be quantified using Huang–Rhys factor *S*. Detailed studies of the *S*‐PLQY relation for a series of 1D hybrid metal halides (C_5_N_2_H_16_Pb_2_Br_6_, C_4_N_2_H_14_PbBr_4_, C_6_N_2_H_16_PbBr_4_, and (C_6_N_2_H_16_)_3_Pb_2_Br_10_) reveal a quantitative structure–property relationship that regulating *S* factor toward 28 leads to the maximum emission.

## Graphical abstract

This work demonstrates a quantitative relationship between photoluminescence quantum yield (PLQY) and exciton–phonon coupling in a series of 1D hybrid metal halides. Using pressure to regulate the exciton–phonon interaction, a near 100% PLQY of broadband emission from self‐trapped excitons is achieved in a corrugated 1D compound C_5_N_2_H_16_Pb_2_Br_6_ whose initial PLQY is 10%.

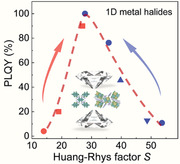

## 1 Introduction

Metal halide perovskites and perovskite‐related materials possess unique lattice and electronic properties that endow them with promising applications in high‐performance photovoltaics and optoelectronics.^[^ ^1^ ^]^ For the low‐dimensional (low‐D) variants, their broadband emission covering the visible‐light spectrum is particularly appealing for the next‐generation solid‐state lighting.^[^ ^2^ ^]^ Various low‐D hybrid metal halides exhibiting broadband emission have been developed in recent years, including 2D (CH_3_CH_2_NH_3_)_4_Pb_3_Br_10−_ *_x_* Cl*_x_*,^[^ ^3^ ^]^ 1D C_4_N_2_H_14_PbBr4,^[^ ^4^ ^]^ and 0D (C_9_NH_20_)_7_(PbCl_4_)Pb_3_Cl_11_.^[^ ^5^ ^]^ Among them, 1D compounds are increasingly attractive as potentially efficient broadband emitters with efficient carrier transport along the chain direction.^[^ ^6^ ^]^ Owing to their quantum confinement and strong electron–phonon coupling, self‐trapped excitons (STEs) could easily form and be stabilized at room temperature,^[^ ^7^ ^]^ resulting the broadband emissions.^[^ ^8^ ^]^ However, the emission efficiencies of 1D metal halides at ambient conditions are still unsatisfactory due to non‐optimal exciton‐phonon coupling. To achieve highly efficient broadband emissions from 1D metal halides, a better fundamental understanding of the luminescence mechanisms and structure–property relationships is needed.

As a thermodynamic variable, pressure provides an effective means to tune the atomic and electronic structures of materials, and subsequently the physical properties.^[^ ^9^ ^]^ In combination with in situ characterization methods, high‐pressure research can further our fundamental understandings of the unique properties in hybrid metal halides. Recently, numerous studies of pressure‐induced and/or enhanced PL in metal halides have been reported,^[^ ^10^ ^]^ while the microscopic mechanisms of the pressure effects are still lively debated. Our recent work has revealed that lattice compression stabilizes the STE states by increasing the exciton binding energy of 1D C_4_N_2_H_14_PbBr_4_, resulting in the enhanced PL efficiency.^[^ ^11^ ^]^ Nevertheless, many questions remain to be answered, for example: 1) Is there an ideal level for STE binding energy or are there other parameters that can evaluate the emission performance better? 2) Is it possible to achieve the ultimate performance of 100% PLQY in 1D metal halides by regulating exciton‐phonon coupling? Addressing these challenges needs suitable material systems with high tunability of both structure and properties as well as advanced regulation and in situ characterization methods.

**Figure 1:**
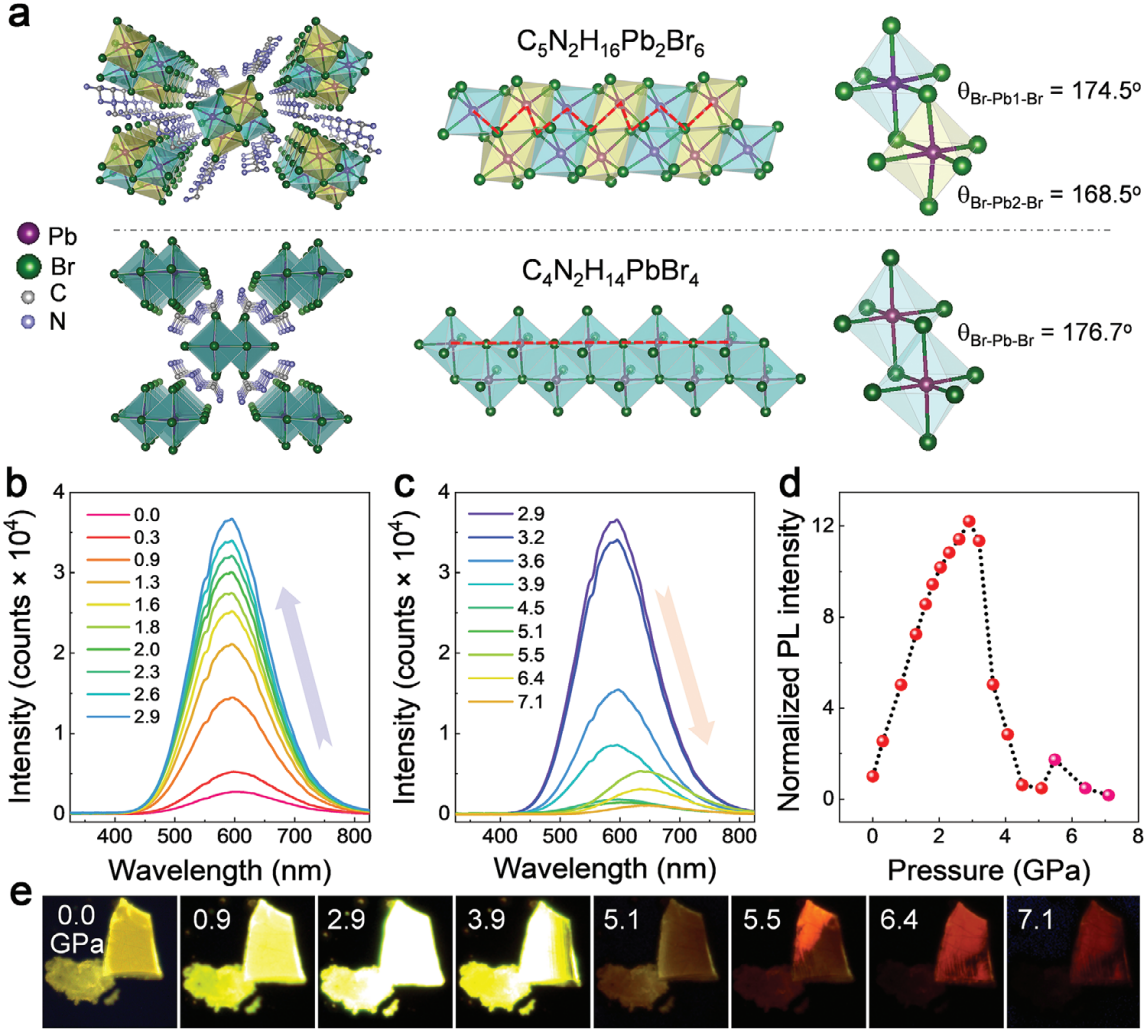
a) Crystal structure of C_5_N_2_H_16_Pb_2_Br_6_ in comparison with C_4_N_2_H_14_PbBr_4_. The left panel shows the overall structures; the middle and right panels indicate the lack of linear —Pb—Br—Pb—Br—Pb— bonding network along the 1D chain direction (red dash line) and the large octahedral distortion in C_5_N_2_H_16_Pb_2_Br_6_, respectively. b,c) Pressure‐induced evolution of emission properties at different pressures. d) The normalized PL integrated intensity as a function of pressure. e) The fluorescent images at selected pressures under UV irradiation (360–390 nm).

In this work, we carefully selected a metal halide C_5_N_2_H_16_Pb_2_Br_6_ with a corrugated 1D structure which permits a high tunability (**Figure** **1**a). Distinguished from other 1D metal halides, this material possesses several unique characteristics, such as a giant structural distortion and strong exciton–phonon coupling which give extremely large STE binding energy of 1.28 eV (vs 0.76 eV for C_4_N_2_H_14_PbBr_4_) and high energy for ground state.^[^ ^4^, ^12^ ^]^ By carefully regulating such a highly‐distorted structure using pressure, here we have reached an unexplored structural region, which offers a rare opportunity to understand the structure–property relationship and also to achieve the ultimate performance. Impressively, significant enhancements in both PL intensity and lifetime were revealed, achieving a near‐unity PLQY at a mild pressure below 3 GPa. By using in situ in‐laboratory and synchrotron‐based characterization methods along with first‐principles calculations, we systematically elucidate the underlying mechanisms of these pressure‐induced dramatic changes in electronic and optical properties. We further establish a quantitative relationship between the PL property and Huang–Rhys factor *S* in a series of 1D metal halides.^[^ ^7^ ^]^ The *S* parameter provides a quantitative description of exciton–phonon coupling that assesses the interactions between STE states and ground states,^[^ ^13^ ^]^ hence is more suitable for evaluating the STE emission performance.

## 2 Results and Discussion

In situ PL spectroscopy and fluorescent imaging were conducted to explore the variations of emission property in the corrugated 1D C_5_N_2_H_16_Pb_2_Br_6_ under high pressure. At ambient conditions, C_5_N_2_H_16_Pb_2_Br_6_ exhibits a broadband emission peaked at 610 nm with a full width at half maximum of 160 nm, which is attributed to the radiative recombination of STEs.^[^ ^12^ ^]^ During compression, the PL intensity of C_5_N_2_H_16_Pb_2_Br_6_ shows a remarkable enhancement and reaches a maximum at 2.9 GPa (Figure 1a). Subsequently, the PL intensity continuously drops from 3 to 5 GPa. Note that an anomalous secondary increase of PL appears at 5.5 GPa (Figure 1c,d). Since the bandgap of C_5_N_2_H_16_Pb_2_Br_6_ also exhibits a notable discontinued drop at a similar pressure range according to the in situ UV–vis absorption spectra (Figure S2, Supporting Information), a pressure‐induced structural change is believed to occur and we will elaborate on it later. Figure 1d shows the normalized PL integrated intensity of C_5_N_2_H_16_Pb_2_Br_6_ as a function of pressure, where the maximum value at 2.9 GPa is more than one order of magnitude higher than that at ambient conditions. In addition, the fluorescent micrographs in Figure 1e exhibit the PL brightness variations with increasing pressure and an obvious emission color change at higher pressures. No obvious difference of emission property can be observed between the single crystal and powder sample of C_5_N_2_H_16_Pb_2_Br_6_ under high pressure. Finally, the emission of C_5_N_2_H_16_Pb_2_Br_6_ gradually vanishes with further compression, and quenches at above 8 GPa (Figure S3, Supporting Information).

**Figure 2:**
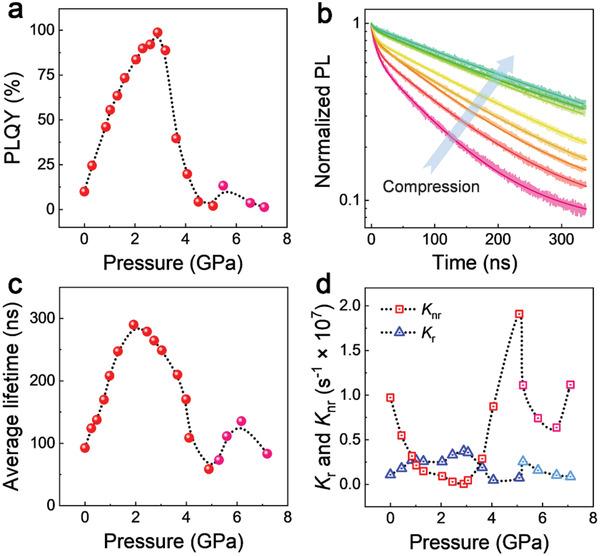
a) The evaluation of PLQY of C_5_N_2_H_16_Pb_2_Br_6_ under high pressure. b) Time‐resolved PL spectra at different pressures. The solid lines are the biexponential fits of the decay dynamics. c) The change of average PL lifetime during compression. d) Pressure dependence of radiative (triangle) and non‐radiative (square) carrier recombination rates.

To assess the high‐pressure PL property more accurately, we further determined the PLQY under the giga‐Pascal pressure scale. Detailed description of the method can be seen in the Supporting Information.^[^ ^11^ ^]^ **Figure** **2**a plots the PLQY of C_5_N_2_H_16_Pb_2_Br_6_ as a function of pressure. Impressively, the PLQY is promoted from the initial value of 10% to the maximum value close to 100% at 2.9 GPa. Besides, the dependence of PL intensity on the excitation power density was examined to rule out the possibility of the emission from defects.^[^ ^3^ ^]^ As shown in Figures S4 and S5, Supporting Information, all the variations at different pressures can be linearly fitted by *I* = *αP*_w_, where *P*_w_ is the excitation laser power and *α* is the intensity coefficient.

Such a significant enhancement of PLQY is related to the variations of atomic and electronic structures of C_5_N_2_H_16_Pb_2_Br_6_. We first traced the change of carrier dynamics which can be revealed by in situ time‐resolved photoluminescence (trPL) spectroscopy. Figure 2b and Figure S6, Supporting Information, show the trPL spectra of C_5_N_2_H_16_Pb_2_Br_6_ at different pressures. We applied biexponential function, *I*(t) = *I*_0_ [*A*_1_·exp(‐*t*/*τ*_1_) + *A*_2_·exp(‐*t*/*τ*_2_)], to fit the PL decay dynamics (Table S1, Supporting Information), where *I*_0_ is the initial PL intensity, *τ*_1_ and *τ*_2_ are the slow‐ and fast‐decay components, respectively, and *A*_1_ and *A*_2_ are the amplitude of two components. The pressure‐dependence average PL lifetime *τ* is calculated by *τ* = (*A*_1_ *τ*_1_^2^ + *A*_2_ *τ*_2_^2^) / (*A*_1_ *τ*_1_+ *A*_2_ *τ*_2_). At ambient conditions, the average lifetime was determined to be 92 ns, which is in line with the reported value.^[^ ^12^ ^]^ During compression, the lifetime enlarges by three times to 270 ns at 2 GPa, and then gradually decreases till 5 GPa where a second enhancement occurs (Figure 2c). Together with the pressure dependence of PLQY, the evolution of radiative (*k*_r_) and non‐radiative (*k*_nr_) recombination rates can be calculated as the average lifetime *τ* is determined by the reciprocal of the sum of radiative (*k*_r_) and non‐radiative (*k*_nr_) recombination rates, and the PLQY is the ratio of the radiative (*k*_r_) recombination rate to the sum of radiative (*k*_r_) and non‐radiative (*k*_nr_) recombination rates (detailed method can be found in the Supporting Information). As shown in Figure 2d, on the one hand, the radiative decay rate increases by three times from 1.08 × 10^6^ s^–1^ at ambient conditions to 3.73 × 10^6^ s^–1^ at 2.9 GPa. On the other hand, impressively, the non‐radiative decay rate is almost completely suppressed at 2.9 GPa, which dominantly contributes to the dramatically enhanced PLQY. In addition, a wavelength‐dependent trPL study on C_5_N_2_H_16_Pb_2_Br_6_ indicates that the broadband emission decay arises from a time‐averaged single ensemble (Figure S7, Supporting Information).^[^ ^14^ ^]^

In situ synchrotron X‐ray diffraction (XRD) measurements were carried out to explore the structural variations of C_5_N_2_H_16_Pb_2_Br_6_ under high pressure (Figure S8, Supporting Information). At ambient conditions, C_5_N_2_H_16_Pb_2_Br_6_ adopts a monoclinic *P*2_1_/c structure with lattice parameters *a* = 7.4746(2) Å, *b* = 11.5691(2) Å, *c* = 20.9689(4) Å, and *β* = 95.405(2)°. The XRD data of C_5_N_2_H_16_Pb_2_Br_6_ were analyzed by Rietveld refinements (Figure S9 and Table S2, Supporting Information) and the detailed analysis and discussion can be seen in the Supporting Information. Briefly, the pressure‐induced variations of lattice constants and unit‐cell volumes are displayed in Figure S10, Supporting Information, where the anisotropic compressibility is due to the different ways of connection in the three directions. By fitting the unit‐cell volume to the Birch–Murnaghan equation of state,^[^ ^15^ ^]^ a volume collapse can be observed, which is a common feature for the isostructural transition.^[^ ^16^ ^]^ The structural changes observed in the XRD results are also reflected in the pressure‐dependent absorption spectra (Figure S2, Supporting Information), suggesting the significant effects of pressure on the atomic and electronic structures. In addition, the obtained electronic structures by density‐functional theory calculations well reproduce the variation trend of experimental bandgaps (Figure S11, Supporting Information).

The broadband emission of 1D hybrid metal halides is originated from the stabilized STEs caused by strong exciton–phonon coupling. The emission efficiency depends profoundly on the depth of STEs, can neither be too shallow nor too deep. That is, if the excitons are easily de‐trapped from the STE states, the STE emission would be inefficient;^[^ ^8^ ^]^ while too deep STE would facilitate the phonon‐assisted non‐radiative recombination which also decreases the emission efficiency.^[^ ^7^ ^]^ The case of C_4_N_2_H_14_PbBr_4_ reported in the previous work belongs to the former scenario;^[^ ^11^ ^]^ in contrast, the case of highly distorted C_5_N_2_H_16_Pb_2_Br_6_ studied herein belongs to the latter scenario, whose STE binding energy is as high as 1.28 eV.^[^ ^12^ ^]^ Huang–Rhys parameter *S* can be used to quantitatively describe the interaction of the STE state and GS and thus could elucidate the underlying mechanisms of pressure‐induced PLQY evolution.^[^ ^1d^ ^]^

**Figure 3:**
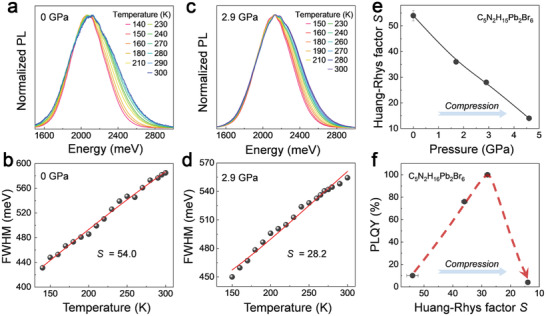
a,c) The temperature‐dependent photoluminescence spectra and b,d) the corresponding fitted values of Huang–Rhys factor *S* for C_5_H_16_N_2_Pb_2_Br_6_ at 0 and 2.9 GPa. FWHM means the full width at half maximum of the PL spectra. e) Pressure‐dependent variations of *S* factor. f) PLQY as a function of Huang–Rhys factor *S*.

As shown in **Figure** **3** and Figure S12, Supporting Information, the *S* values of C_5_N_2_H_16_Pb_2_Br_6_ are determined by fitting the temperature‐dependent PL property under various pressures. The detailed experimental description can be seen in the Supporting Information. The *S* parameter decreases monotonously from 54 at ambient pressure to 28 at 2.7 GPa (Figure 3e). We plotted the PLQY as a function of *S* parameter in Figure 3f. PLQY increases significantly with the decrease of *S* during compression, reaches the maximum at *S* = 28, and then decreases with a further decrease of *S* at higher pressures. The variation profile suggests an optimized *S* value that would lead to the highest PLQY of C_5_N_2_H_16_Pb_2_Br_6_. As far as we know, such a relationship has never been observed due to the limited tunability of other 1D metal halides. The highly distorted structure of C_5_N_2_H_16_Pb_2_Br_6_ enables to reach an otherwise unexplorable structural region, thus, provides more tuning possibilities for the better fundamental understanding and high performance (100% PLQY).

**Figure 4:**
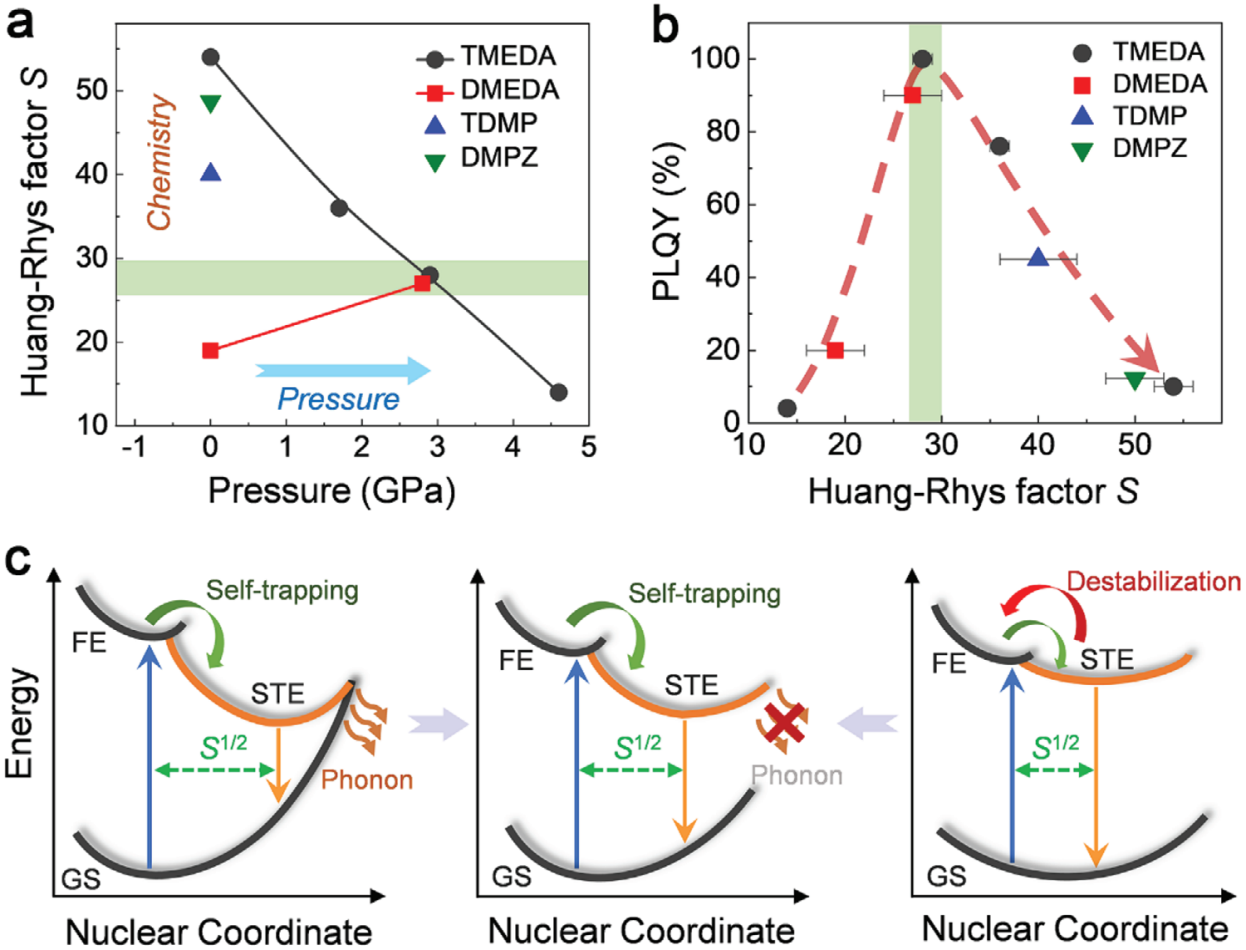
a) Huang–Rhys factor *S* values of various 1D hybrid metal halides at different pressures. TMEDA, DMEDA, TDMP, and DMPZ indicate C_5_N_2_H_16_Pb_2_Br_6_, C_4_N_2_H_14_PbBr_4_, C_6_N_2_H_16_PbBr_4_, and (C_6_N_2_H_16_)_3_Pb_2_Br_10_, respectively. b) The relationship between PLQY and *S* factor in 1D hybrid metal halides. c) Schematic illustration of the influence of the regulated *S* factor on exciton dynamics and emission property in the 1D hybrid metal halides (FE, free exciton state; GS, ground state; STE, self‐trapped exciton state; *S*, Huang–Rhys factor). Strong phonon‐assisted non‐radiative decay exists when *S* is overly large (left panel, for the case of C_5_N_2_H_16_Pb_2_Br_6_ at ambient pressure); while too small *S* value destabilizes the STE states and weakens the broadband emission (right panel, for the case of C_4_N_2_H_14_PbBr_4_ at ambient pressure). By tuning the Huang–Rhys factor *S* to a suitable value, the maximum emission can be achieved (middle panel).

The close relationship between the Huang–Rhys factor *S* and the STE emission efficiency can be further demonstrated in the 1D C_4_N_2_H_14_PbBr_4_, which has a less distorted structure and relatively low STE binding energy at ambient conditions (0.76 eV). Pressure promotes the broadband emission of C_4_N_2_H_14_PbBr_4_ by stabilizing the STE states, that is, increasing *S*. The *S* values were determined to be 19 and 27 at ambient pressure and 2.8 GPa (**Figure** **4**a; Figure S13, Supporting Information), respectively. We plotted the *S* values and corresponding PLQY (20% and 90%) into Figure 4b, intriguingly, their relationship follows the same curve as revealed in C_5_N_2_H_16_Pb_2_Br_6_. The consistent *S*‐PLQY relationship revealed for both C_5_N_2_H_16_Pb_2_Br_6_ and C_4_N_2_H_14_PbBr_4_ confirms the existence of an optimal exciton–phonon interaction, which leads to the highest PLQY in these 1D hybrid metal halides. It is worth noting that pressure has different effects on regulating the exciton–phonon coupling of these two material systems. Here we have identified that the governing parameter is not the pressure, but the Huang–Rhys factor *S* which is closely related to structural distortion and exciton–phonon coupling.

We further noticed the recently developed 1D metal halides C_6_N_2_H_16_PbBr_4_ with a PLQY of 45% and (C_6_N_2_H_16_)_3_Pb_2_Br_10_ with a PLQY of 12% at ambient conditions.^[^ ^17^ ^]^ Their *S* values were also determined by fitting the temperature‐dependent PL property (Figure S14, Supporting Information). We plotted the *S* values and corresponding PLQYs of 45% and 12% into Figure 4b. Impressively, the *S*‐PLQY relationship of all these 1D hybrid metal halides collapse on the same curve, that regulating the Huang–Rhys factor *S* toward ≈28 leads to the highest PLQY.

Huang–Rhys parameter *S* could, therefore, serve as the figure of merit for evaluating and optimizing the STE emission that the ideal *S* value should be intermediate. On the one hand, The highly distorted structure and strong exciton‐phonon coupling (with exciton binding energy of 1.28 eV) in C_5_N_2_H_16_Pb_2_Br_6_, expressed as the overly large *S*, rise the GS energy and thus lead to the crossover between STE state and GS as shown in the left panel of Figure 4c.^[^ ^7^ ^]^ Consequently, the trapped excitons can easily annihilate by the phonon‐assisted pathway, resulting in a high non‐radiative recombination rate at ambient conditions. This is unfavorable for the efficient emission of STEs. With compression, the wavefunction overlap between the STE state and the GS reduces caused by the pressure‐regulated exciton–phonon interaction (middle panel of Figure 4c), and therefore the phonon‐assisted non‐radiative decay of STEs is greatly suppressed (Figure 2d), giving rise to the high PLQY. On the other hand, the much weaker exciton–phonon coupling in C_4_N_2_H_14_PbBr_4_, expressed as a much smaller *S* at ambient pressure, brings unstable STEs and thus inefficient emission (right panel of Figure 4c). In this case, pressure promotes the broadband emission by stabilizing the STE states toward a suitable *S* value.

## 3 Conclusion

Using pressure to regulate the exciton–phonon coupling, a near‐unity PLQY of STE‐based broadband emission was obtained in a corrugated 1D hybrid metal halide C_5_N_2_H_16_Pb_2_Br_6_. The high tunability of this material enables to realize an otherwise unattainable feature for both property optimization and mechanism understanding. Compression effectively modulates the overly strong exciton–phonon interaction of C_5_N_2_H_16_Pb_2_Br_6_ to a suitable level and reduces the overlap between the STE states and the ground state, leading to the suppressed phonon‐assisted non‐radiative loss and thus the tenfold enhanced emission. By quantifying the exciton–phonon coupling using Huang–Rhys factor *S*, we have uncovered a quantitative relationship that regulating *S* factor toward 28 leads to the highest PLQY of this 1D metal halide. More importantly, the *S*–PLQY relationship of other 1D metal halides including C_4_N_2_H_14_PbBr_4_, C_6_N_2_H_16_PbBr_4_, and (C_6_N_2_H_16_)_3_Pb_2_Br_10_ follows the same curve, which further verifies the rationality of the newly revealed principle. This work not only achieves the ultimate performance of 100% PLQY in 1D hybrid metal halide but also quantitatively demonstrates the relationship between the exciton–phonon coupling and STE emission.

## Conflict of Interest

The authors declare no conflict of interest.

## Supporting information

Supporting Information

## Data Availability

Research data are not shared.

## References

[1] a) H. J. Snaith , Nat. Mater. 2018, 17, 372; 29686248 10.1038/s41563-018-0071-z

[2] a) M. I. Saidaminov , O. F. Mohammed , O. M. Bakr , ACS Energy Lett. 2017, 2, 889;

[3] E. R. Dohner , A. Jaffe , L. R. Bradshaw , H. I. Karunadasa , J. Am. Chem. Soc. 2014, 136, 13154. 25162937 10.1021/ja507086b

[4] Z. Yuan , C. Zhou , Y. Tian , Y. Shu , J. Messier , J. C. Wang , L. J. van de Burgt , K. Kountouriotis , Y. Xin , E. Holt , K. Schanze , R. Clark , T. Siegrist , B. Ma , Nat. Commun. 2017, 8, 14051. 28051092 10.1038/ncomms14051 PMC5216108

[5] C. Zhou , H. Lin , M. Worku , J. Neu , Y. Zhou , Y. Tian , S. Lee , P. Djurovich , T. Siegrist , B. Ma , J. Am. Chem. Soc. 2018, 140, 13181. 30230822 10.1021/jacs.8b07731

[6] T. Liu , W. Tang , S. Luong , O. Fenwick , Nanoscale 2020, 12, 9688. 32319990 10.1039/d0nr01495h

[7] S. Li , J. Luo , J. Liu , J. Tang , J. Phys. Chem. Lett. 2019, 10, 1999. 30946586 10.1021/acs.jpclett.8b03604

[8] K. S. Song , R. T. Williams , Self‐Trapped Excitons, vol. 105, Springer, Berlin Heidelberg 2013.

[9] a) X. Lu , W. Yang , Z. Quan , T. Lin , L. Bai , L. Wang , F. Huang , Y. Zhao , J. Am. Chem. Soc. 2014, 136, 419; 24320708 10.1021/ja410810w

[10] a) S. Guo , Y. Zhao , K. Bu , Y. Fu , H. Luo , M. Chen , M. P. Hautzinger , Y. Wang , S. Jin , W. Yang , X. Lü , Angew. Chem., Int. Ed. 2020, 59, 17533; 10.1002/anie.202001635 32627251

[11] Y. Wang , S. Guo , H. Luo , C. Zhou , H. Lin , X. Ma , Q. Hu , M. H. Du , B. Ma , W. Yang , X. Lu , J. Am. Chem. Soc. 2020, 142, 16001. 32870668 10.1021/jacs.0c07166

[12] H. Lin , C. Zhou , J. Neu , Y. Zhou , D. Han , S. Chen , M. Worku , M. Chaaban , S. Lee , E. Berkwits , T. Siegrist , M. H. Du , B. Ma , Adv. Opt. Mater. 2019, 7, 1801474.

[13] a) K. Huang , A. Rhys , Proc. R. Soc. London, Ser. A 1950, 204, 406;

[14] J. E. Thomaz , K. P. Lindquist , H. I. Karunadasa , M. D. Fayer , J. Am. Chem. Soc. 2020, 142, 16622. 32909430 10.1021/jacs.0c05636

[15] F. Birch , Phys. Rev. 1947, 71, 809.

[16] S. Wang , J. Zhu , Y. Zhang , X. Yu , J. Zhang , W. Wang , L. Bai , J. Qian , L. Yin , N. S. Sullivan , C. Jin , D. He , J. Xu , Y. Zhao , Proc. Natl. Acad. Sci. U. S. A. 2015, 112, 15320. 26604314 10.1073/pnas.1510415112 PMC4687540

[17] a) L. Mao , P. Guo , M. Kepenekian , I. Hadar , C. Katan , J. Even , R. D. Schaller , C. C. Stoumpos , M. G. Kanatzidis , J. Am. Chem. Soc. 2018, 140, 13078; 30212624 10.1021/jacs.8b08691

