# Supplementary material for: Regulating Exciton–Phonon Coupling to Achieve a Near‐Unity Photoluminescence Quantum Yield in One‐Dimensional Hybrid Metal Halides
Source: Adv Sci (Weinh). 2021 May 22;8(14):2100786. doi: 10.1002/advs.202100786 (PMC8292847; doi:10.1002/advs.202100786)
Supplement: Supplementary file 1 — Supporting Information [file ADVS-8-2100786-s001.pdf]

© 2021 Wiley-VCH GmbH

ADVANCED SCIENCE
Open Access

Supporting Information

for *Adv. Sci.,* DOI: 10.1002/advs.202100786

Regulating exciton-phonon coupling to achieve a near-unity photoluminescence quantum yield in one-dimensional hybrid metal halides

*Hui Luo, Songhao Guo,Yubo Zhang, Kejun Bu, Haoran Lin, Yingqi Wang, Yanfeng Yin, Dongzhou Zhang, Shengye Jin, Wenqing Zhang, Wenge Yang, Biwu Ma, and Xujie Lü**

Supporting Information

Regulating exciton-phonon coupling to achieve a near-unity photoluminescence quantum yield in one-dimensional hybrid metal halides

*Hui Luo, Songhao Guo,Yubo Zhang, Kejun Bu, Haoran Lin, Yingqi Wang, Yanfeng Yin, Dongzhou Zhang, Shengye Jin, Wenqing Zhang, Wenge Yang, Biwu Ma, and Xujie Lü**

H. Luo, Dr. K. Bu, S. Guo, Dr. Y, Wang, Prof. W. Yang, Prof. X. Lü
Center for High Pressure Science and Technology Advanced Research (HPSTAR), Shanghai 201203, China
E-mail: xujie.lu@hpstar.ac.cn

Prof. Y. Zhang, Prof. W. Zhang
Department of Physics and Shenzhen Institute for Quantum Science & Engineering, Southern University of Science and Technology, Shenzhen, Guangdong 518055, China

Dr. H. Lin
Hoffmann Institute of Advanced Materials, Shenzhen Polytechnic, Shenzhen, Guangdong 518055, China

Dr. D. Zhang
Hawaii Institute of Geophysics & Planetology, University of Hawaii Manoa, Honolulu, Hawaii 96822, USA

Y. Yin, Prof. S. Jin
State Key Laboratory of Molecular Reaction Dynamics and Dynamics Research Center for Energy and Environmental Materials, Dalian Institute of Chemical Physics, Chinese Academy of Sciences, Dalian, Liaoning 116023, China

Prof. B. Ma
Department of Chemistry and Biochemistry, Florida State University, Tallahassee, FL 32306, USA

Experiment details

Sample preparation

Lead (II) bromide (400 mg, 1.09 mmol) and $C_5N_2H_{16}Br_2$ (100 mg, 0.379 mmol) were mixed at 1:0.35 molar ratio and dissolved in hydrobromic acid (3 mL) to form a clear precursor solution. Bulk crystals were prepared by diffusing ethyl ether (2 mL) into the prepared precursor solution (1 mL) at room temperature for overnight. The large colorless crystals were washed with ethyl ether and dried under reduced pressure.

In situ high-pressure characterizations

The high-pressure environment was provided by symmetrical diamond anvil cells (DACs). Type II-a ultralow fluorescence diamonds with a culet size of 300 μm were used. The high-pressure sample chamber was formed from a stainless-steel gasket with a pre-indented thickness of about 50 μm and a hole with a diameter of about 150 μm by laser-drilling the center part of it. One $C_5N_2H_{16}Pb_2Br_6$ single crystal (~ 40 × 80 μm) and a ruby ball (for pressure measurements) were loaded inside the sample chamber, and the pressures were monitored by the ruby fluorescence method.[1] Mineral oil was used as the pressure transmitting medium.

The PL spectra, absorption spectra, optical images, and fluorescent images were measured in a home-designed spectroscopy system (Gora-UVN-FL, assembled by Ideaoptics, Shanghai, China). PL spectra were measured using a 360 nm excitation laser at a power of 10 mW. UV-Vis absorption spectra were collected using a Xenon light source with wavelength range between 200 nm and 1700 nm. The PL spectrum were center and integrated intensity were fitted with standard Gaussian function. *In situ* Raman spectra were collected a home-designed Raman spectrometer with a 532.1 nm excitation laser at GSECARS, APS, ANL.

We used a home-built time-correlated single-photon counting (TCSPC) module to measure the time-resolved PL spectra. Excitation of the sample is achieved with a pulse laser (PIXEA-CU-1, AUREA, France) of 375nm, 5 MHz repetition rate, and ~35ps pulse width.

The *in-situ* high pressure X-ray diffraction experiments were performed at beamline 13 BM-C of GeoSoilEnviroCARS at the Advanced Phonon Source (APS), Argonne National Laboratory (ANL). The wavelength of the monochromatic X-ray beam is 0.4340 Å. The diffraction patterns were collected by a Pilatus 1M imaging plate and integrated by the Dioptas software.[2] Structure refinements for low- and high-pressure phases were carried out by the Rietveld method, using GSAS-II software.[3]

Computational details

The density functional theory calculations are performed using the Vienna *Ab initio* Simulation Package (VASP).[4] We use the exchange-correlation functional in the form of Perdew-Burke-Ernzerhof (PBE),[5] and the van der Waals interaction is further included using the optB86b-vdW functional.[6] Spin-orbit coupling is also considered in the electronic structure calculations. The energy cutoff of the plane waves is 500 eV, and the K-mesh is $6 \times 4 \times 2$ for calculating the electronic properties. All the calculations are based on the experimental crystal structures.

Detailed discussion

Experimental observation of bandgap under high pressure

$C_5N_2H_{16}Pb_2Br_6$ crystal has been reported to be an indirect band structure at ambient conditions.[7] Thus, an indirect bandgap Tauc plot was used to calculate the bandgap values by extrapolating the linear portion of $(\alpha)^{1/2}$ versus hv, where α is the absorption coefficient, hv is the photon energy. The bandgap of $C_5N_2H_{16}Pb_2Br_6$ crystal is 3.36 eV at ambient conditions

and continuously decreases during compression. The color of $C_5N_2H_{16}Pb_2Br_6$ crystal changes from transparency to light yellow, as shown in the optical micrographs (**Figure S2b**). A drop observed at around 5 GPa indicates a structure change in $C_5N_2H_{16}Pb_2Br_6$ (**Figure S2c**), which is in line with the discontinued PL variation at similar pressure.

Calculation of PLQY under high pressure

Because of the constant excitation intensity and corresponding absorbance at different pressures, the pressure dependence of PLQY can be correlated with PL intensity using the following equation,[8]

$$\Phi = \Phi_0 \frac{\int F(\lambda_{em})}{\int F_0(\lambda_{em})} \frac{A_0(\lambda_{ex})}{A(\lambda_{ex})} \frac{n^2}{{n_0}^2} \tag{1}$$

where Φ stands for PLQY, $\int F(\lambda_{em})$ is the integrated intensity of emission, $A(\lambda_{ex})$ is the absorption at the excitation wavelength, n is the refractive index, and n_0 is the initial refractive index at ambient conditions which has been measured to be 1.4. The correction factor (n^2/n_0^2) is derived from a point source, and the use of this ratio is found to be valid for many detector geometries, n could be estimated from Clausius-Messitte equation and Lorentz-Lorenz equation,[9]

$$\frac{n^2 - 1}{n^2 + 2} \cdot \frac{1}{\rho} = \frac{4\pi}{3} \cdot N_A \cdot \alpha = R_{LL} \tag{2}$$

where density ρ could be calculated from cell volume, R_{LL} is Lorentz-Lorenz constant which is related to polarizability α. For ionic crystals, the change of R_{LL} under pressure is generally small due to the closed shell structures. The variation of density can be calculated from the XRD refinement.

Calculation of K_r and K_{nr} under high pressure

From the PLQY and lifetime, we could solve the radiative and non-radiative recombination rate at each pressure point by[8b, 10]

$$\Phi = \frac{k_r}{k_r + k_{nr}} \text{ and } \tau = \frac{1}{k_r + k_{nr}} \quad (3)$$

(Φ stands for PLQY, K_r stands for radiative carrier recombination rate, K_{nr} stands for nonradiative carrier recombination rate, τ stands for an average lifetime).

In situ high-pressure XRD analysis and discussion

Synchrotron X-ray diffractions (XRD) were carried out to understand the structural origin under high pressure. At ambient conditions, $C_5N_2H_{16}Pb_2Br_6$ adopts a monoclinic $P2_1/c$ structure with lattice parameters a = 7.4746(2) Å, b = 11.5691(2) Å, c = 20.9689(4) Å, and β = 95.405(2)°. **Figure S7a** shows the view of crystal structure. Along the a-axis, the octahedra are connected through non-coplanar edges to form the first 1D zigzag chain, then the second chain slide and stack together with the first chain, connected through edge-sharing. The corrugated double chain metal halide nanowires $[Pb_2Br_6]^{2-}$ are separated by the organic cations $[C_5N_2H_{16}]^{2+}$ (N, N, N'-trimethylethylene-diammonium, $TMEDA^{2+}$). **Figure S7b** shows the integrated XRD patterns at select pressures. With increasing pressure, all Bragg diffraction peaks gradually broaden and continuously shift to the large 2θ direction due to the lattice contraction. Only the relative intensities of several peaks change up to 5 GPa (as marked in **Figure S7**) without any peak's appearance or disappearance, which could signify an isostructural transition.[11] Under higher pressures, a certain degree of structural disorder sets in. The XRD data were analyzed by Rietveld refinements (**Figure S8 and Table S2**) and the $P2_1/c$ space group was indexed under different pressures with an isostructural phase transition. The variations of lattice constants and unit-cell volumes of $C_5N_2H_{16}Pb_2Br_6$ under high pressure are displayed in **Figure S9a and S9b.** The anisotropic compressibility is due to the different ways of connection in the three directions. In the low-pressure phase (LP), the

compressibility of the *c*-axis is larger than that of the *b*-axis because of the edge-sharing between two chains, and the compressibility of 1D chain direction of *a*-axis is the smallest due to the connection through inorganic octahedra. After the transition, the compressibility of *a*-axis is higher in the high-pressure phase (HP) which may arise from the octahedral tilting in the corrugated 1D chain along *a*-axis. By fitting the unit-cell volume to the Birch-Murnaghan equation of state (see the followings), a volume collapse can be observed, which is a common feature for the isostructural transition.[12]

Fitting cell volume data to the Birch-Murnaghan equation of state,[13]

$$P(V) = \frac{3K_0}{2}\left[\left(\frac{V_0}{V}\right)^{\frac{7}{3}} - \left(\frac{V_0}{V}\right)^{\frac{5}{3}}\right]\left\{1 + \frac{3}{4}(K' - 4)\left[\left(\frac{V_0}{V}\right)^{\frac{2}{3}} - 1\right]\right\} \quad (4)$$

where V_0, V are the volume at ambient and high pressure, respectively; K_0 is the bulk modulus, and K' is the derivative of the bulk modulus with respect to pressure. The bulk modulus K_0 of the low-pressure phase is estimated to be about 14.7 GPa with K' fitted at 8.9. When the pressure exceeded 5.13 GPa, K_0 is estimated to be about 41.2 GPa with K' at fixed value 4. The higher value of K_0 in the high-pressure phase suggests that it is more difficult to be compressed compared with the low-pressure phase.

Determination of Huang-Rhys factor *S* values

In principle, the Huang-Rhys factor (*S*) reflects the coupling strength between electrons and phonons. A large Huang-Rhys factor *S* means easy formation of STE states which is necessary for effective broadband emission.

On the other hand, the Huang-Rhys factor factor *S* has a positive correlation with the coordinate difference between the free-exciton and STE configurations, ΔQ, which can be described as the following equation:[14]

$$S = \frac{1}{2}\frac{M\omega}{\hbar}(\Delta Q)^2$$

Where M is the reduced mass of the vibrating system, ω is phonon frequency, $\hbar$ is the reduced Planck constant.

An overly large ΔQ (or S) will lead to the tendency for the excited- and ground-state curves to cross in the configuration coordinate diagram, which means that some excited electrons and holes will recombine via this interaction non-radiatively, emitting several phonons.[15] Thereby, the Huang-Rhys factor S value could potentially serve as the figure of merit for the design of efficient emission from STEs. The ideal value of the Huang-Rhys parameter should be intermediate for efficient STE emission.[15b]

The Huang-Rhys factor S can be obtained by fitting the temperature-dependent full-width at half-maxima (FWHM) of photoluminescence peaks using the following equation.[16]

$$FWHM = 2.36\sqrt{S}\hbar\omega\sqrt{\cot\left(\frac{\hbar\omega}{2k_BT}\right)}$$

where ω is phonon frequency, k_B is the boltzmann constant, and T is temperature.

On the other hand, if the temperature-dependent photoluminescence energy shows obvious shifts, the S values can be better determined using the following equation:[17]

$$E_p(T) = E_p(0) + S\hbar\omega\left[\cot\left(\frac{\hbar\omega}{2k_BT}\right) - 1\right]$$

where $E_p(T)$ is the temperature-dependence PL energy, $E_p(0)$ is the PL energy at zero temperature. Note that the Huang-Rhys factor S values fitted by these two equations has demonstrated to be consistent and comparable, and also in good agreement with the theoretically simulated results.[18] Based on the above discussion, the Huang-Rhys factors of $C_5N_2H_{16}Pb_2Br_6$, $C_6N_2H_{16}N_2PbBr_4$, $C_4N_2H_{14}PbBr_4$, $(C_6N_2H_{16})_3Pb_2Br_{10}$ at various pressures were determined, as shown in **Figure S12, S13 and S14**.

Supplementary Figures

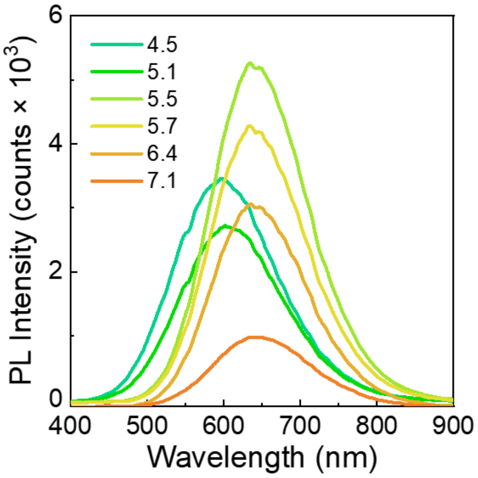

Figure S1. The PL spectra of $C_5N_2H_{16}Pb_2Br_6$ at high pressures beyond 4.5 GPa.

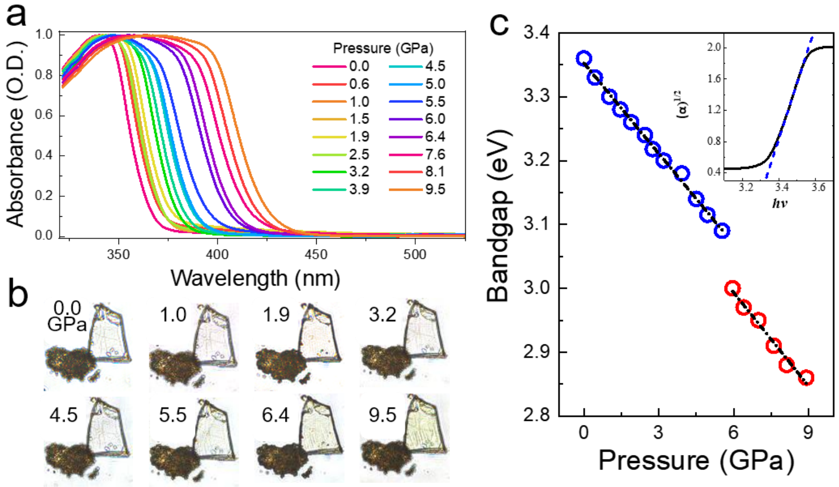

Figure S2. (a) UV-Vis absorption spectra and (b) optical images of $C_5N_2H_{16}Pb_2Br_6$ under high pressure. (c) The change of bandgap as a function of pressure and the Tauc plot fitting for the UV-Vis spectrum of $C_5N_2H_{16}Pb_2Br_6$ at ambient conditions.

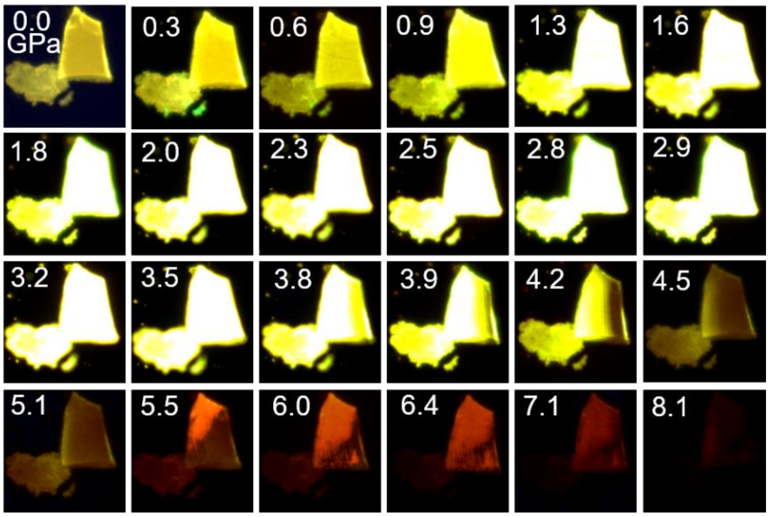

Figure S3. PL micrographs of $C_5N_2H_{16}Pb_2Br_6$ crystal under high pressure. Part of these results at selected pressures are displayed in Figure 1d in the main text (the position of PL measurement was changed when the isostructural phase transition occurred).

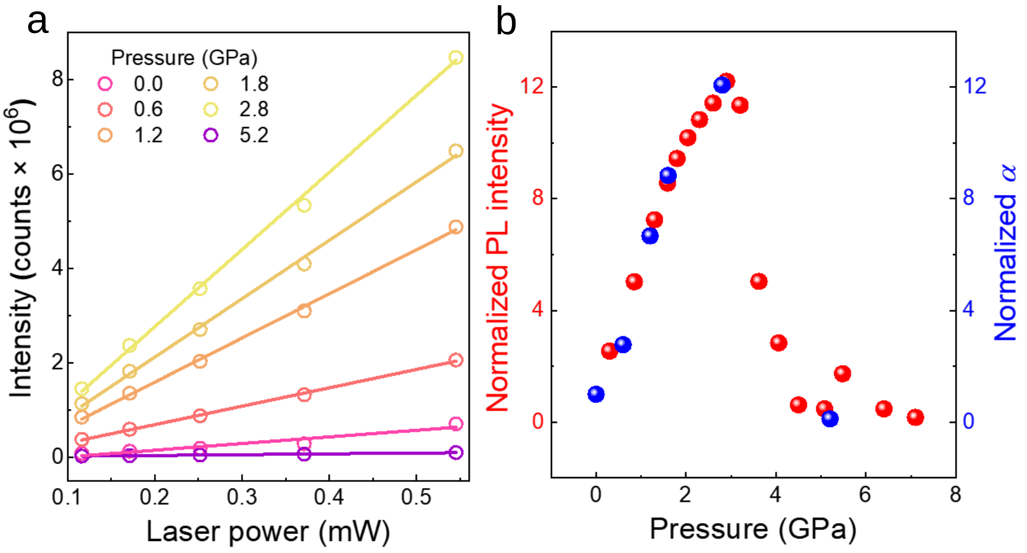

Figure S4 (a) Power-law fittings of power-dependent PL intensity of $C_5N_2H_{16}Pb_2Br_6$ at different pressures. **(b)** The evaluation of α compared with PL intensity under high pressure. The parameter of α can also be used to estimate the emission efficiency, thus we have added the pressure-dependent evolution of α in Figure S4b. The intensity coefficient α exhibits 12-fold enhancement at 2.8 GPa compared with that at ambient conditions which is in line with the evolutions of pressure-dependent PL intensity.

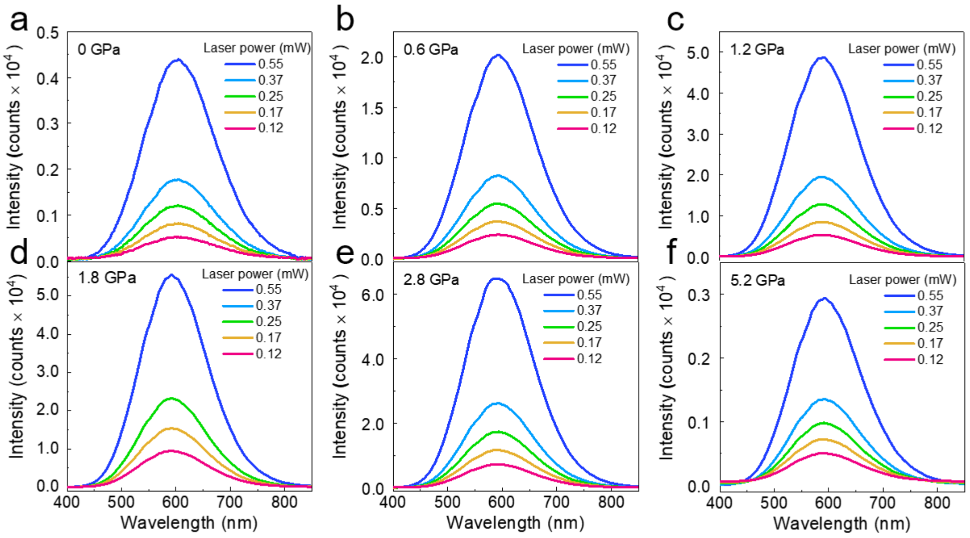

Figure S5. A series of excitation power-dependent PL spectra of $C_5N_2H_{16}Pb_2Br_6$ under high pressure.

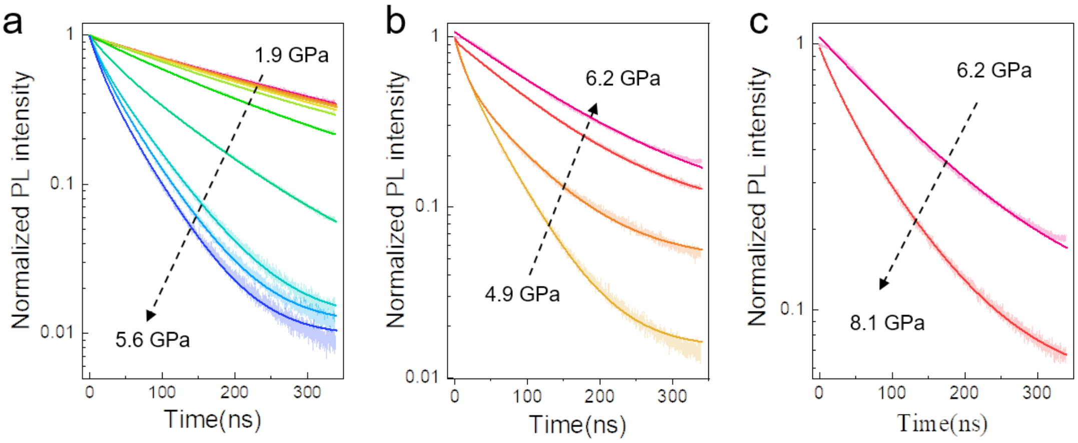

Figure S6. The trPL spectra and corresponding fitting curves of $C_5N_2H_{16}Pb_2Br_6$ at different pressures.

Table S1. The fitting parameters of trPL results and calculated average PL lifetime.

A_1	τ_1	A_2	τ_2	$\tau_{(average)}$	Pressure (GPa)
0.303	8.61125	0.5760	96.53193	92.59710	0
0.269	6.97004	0.6330	126.86143	124.12273	0.3
0.169	6.21476	0.7274	139.02330	137.65724	0.5
0.205	6.02957	0.7076	171.53406	169.86716	0.8
0.170	5.95753	0.7511	209.48961	208.18713	1.0
0.081	6.23634	0.8193	247.98686	247.38811	1.3
0.061	6.70557	0.8052	248.35082	247.85986	1.6
0.030	5.59154	0.8830	290.20061	290.01728	1.9
0.028	5.85499	0.8788	279.13041	278.94795	2.5
0.011	5.02489	0.8817	264.63620	264.57276	2.7
0.018	5.04586	0.8673	249.06921	248.96619	3.0
0.008	11.69472	0.8913	248.67461	244.45094	3.3
0.068	50.25255	0.8637	195.50193	170.45989	4.0
0.241	32.30392	0.7304	115.84341	108.79220	4.1
0.271	12.13767	0.6994	53.85244	50.49955	4.8
0.389	13.70361	0.5309	80.68524	73.26516	5.3
0.043	2.84594	0.8534	111.53925	111.39811	5.6
0.489	135.4432	0.4850	135.44567	135.44446	6.2
0.225	30.35745	0.6913	91.61289	85.65484	6.8
0.353	36.58969	0.5790	94.25697	83.23135	7.2
0.407	13.30881	0.5116	64.60712	57.38897	7.8
0.564	9.31821	0.4028	43.76672	35.85044	8.1

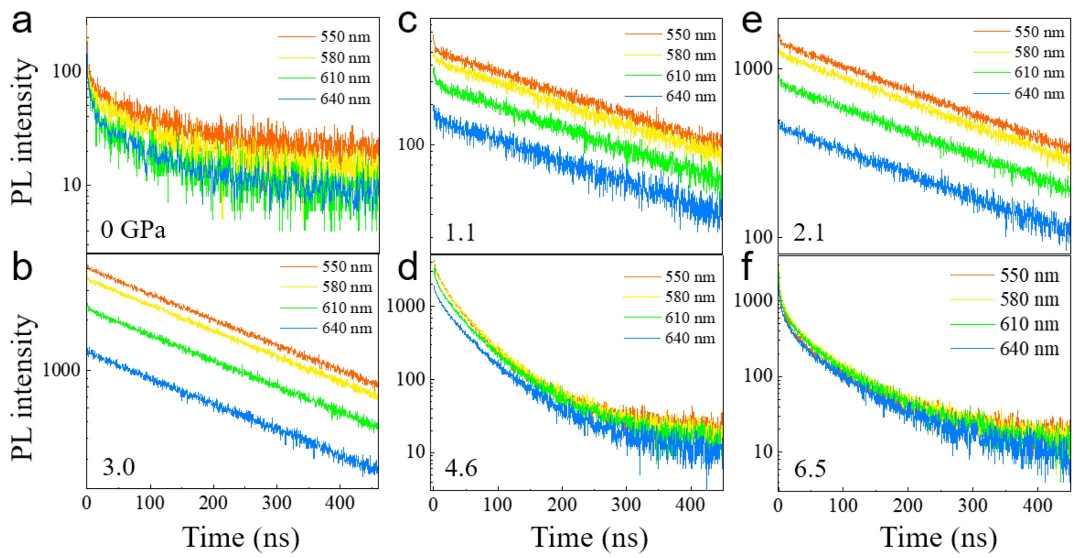

Figure S7. Luminescent emission at representative wavelengths. Each decay was collected for identical periods of time under the same experimental conditions (the ordinate is in logarithmic). Compared with prior works studying the PL decay times point to a wavelength dependence of the emission, the time-averaged single ensemble in $C_5N_2H_{16}Pb_2Br_6$ is likely caused by spectral diffusion throughout the inhomogeneously broadened excited-state spectrum that is fast compared to the PL decay.

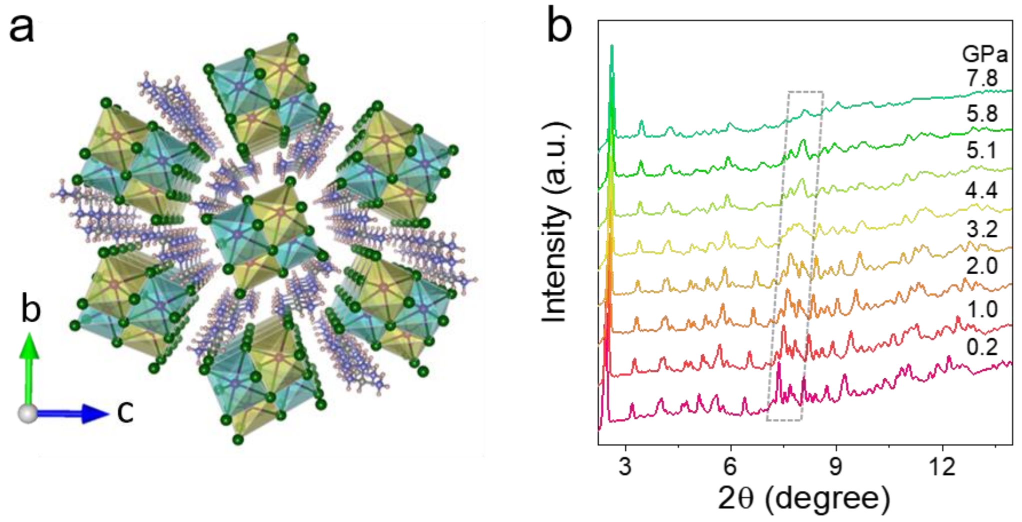

Figure S8. (a) Crystal structure at ambient conditions along *a*-axis. (b) Synchrotron radiation XRD spectra of 1D perovskite under pressure and synchrotron radiation XRD spectra of $C_5N_2H_{16}Pb_2Br_6$ under pressure.

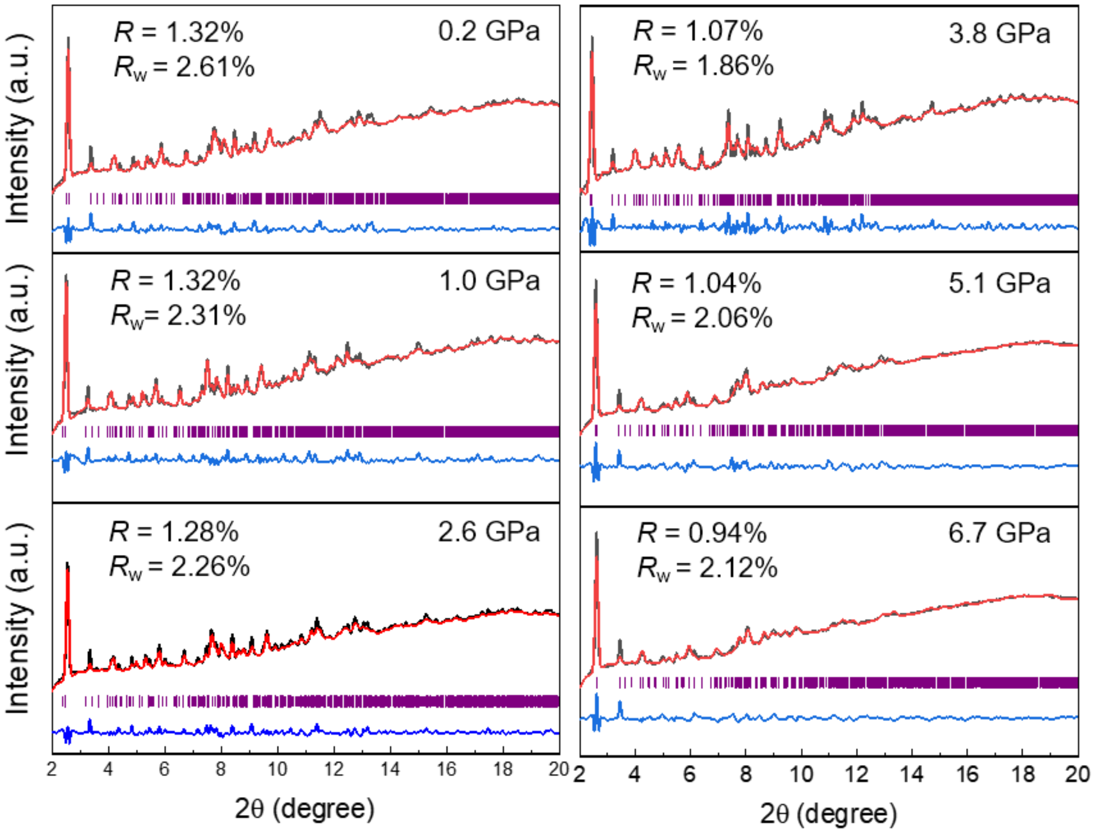

Figure S9. Rietveld refinements at 0.2, 1.0, 2.6, 3.8, 5.1, and 6.7 GPa for $C_5N_2H_{16}Pb_2Br_6$ powder.

Table S2. The fitting parameters of ***a, b, c, β, R,*** and $\boldsymbol{R_w}$ at different pressures

a **(Å)**	***b*** **(Å)**	***c*** **(Å)**	***β***	***R*** **(%)**	$\boldsymbol{R_w}$ **(%)**	**Pressure (GPa)**
7.542	11.676	21.119	95.365	1.32	2.61	0.2
7.504	11.618	20.935	95.374	1.32	2.31	0.4
7.458	11.538	20.755	95.363	1.28	2.26	0.8
7.428	11.485	20.631	95.381	1.17	1.98	1.0
7.378	11.375	20.396	95.347	1.40	2.54	1.6
7.336	11.316	20.261	95.408	1.29	2.33	2.0
7.299	11.251	20.145	95.444	1.13	2.06	2.6
7.262	11.193	20.014	95.477	1.13	1.88	3.3
7.222	11.142	19.918	95.506	1.07	1.86	3.8
7.202	11.161	19.773	95.450	1.08	2.09	4.4
7.168	11.200	19.296	95.241	1.04	2.06	5.1
7.126	11.169	19.195	95.282	0.95	1.86	5.8
7.096	11.083	19.179	95.665	0.94	2.12	6.7
7.069	11.019	19.115	95.673	0.89	1.83	7.8

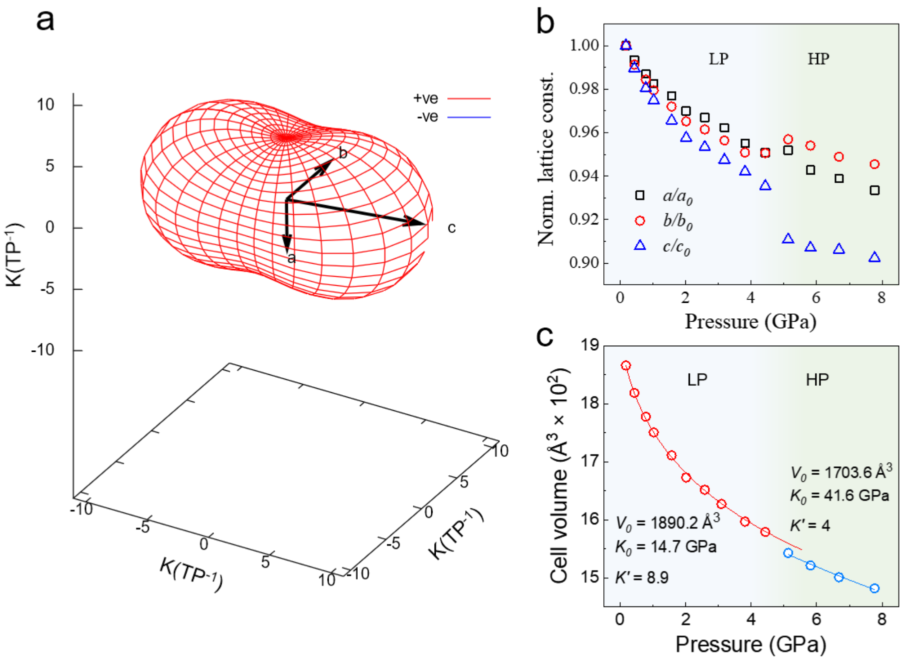

Figure S10. (a) Compressibility indicatrix of $C_5N_2H_{16}Pb_2Br_6$. It is obvious that the compression of *c* axis is visibly stronger than that of *a* and *b* axes. (b, c) Pressure dependence of unit cell parameters and volume.

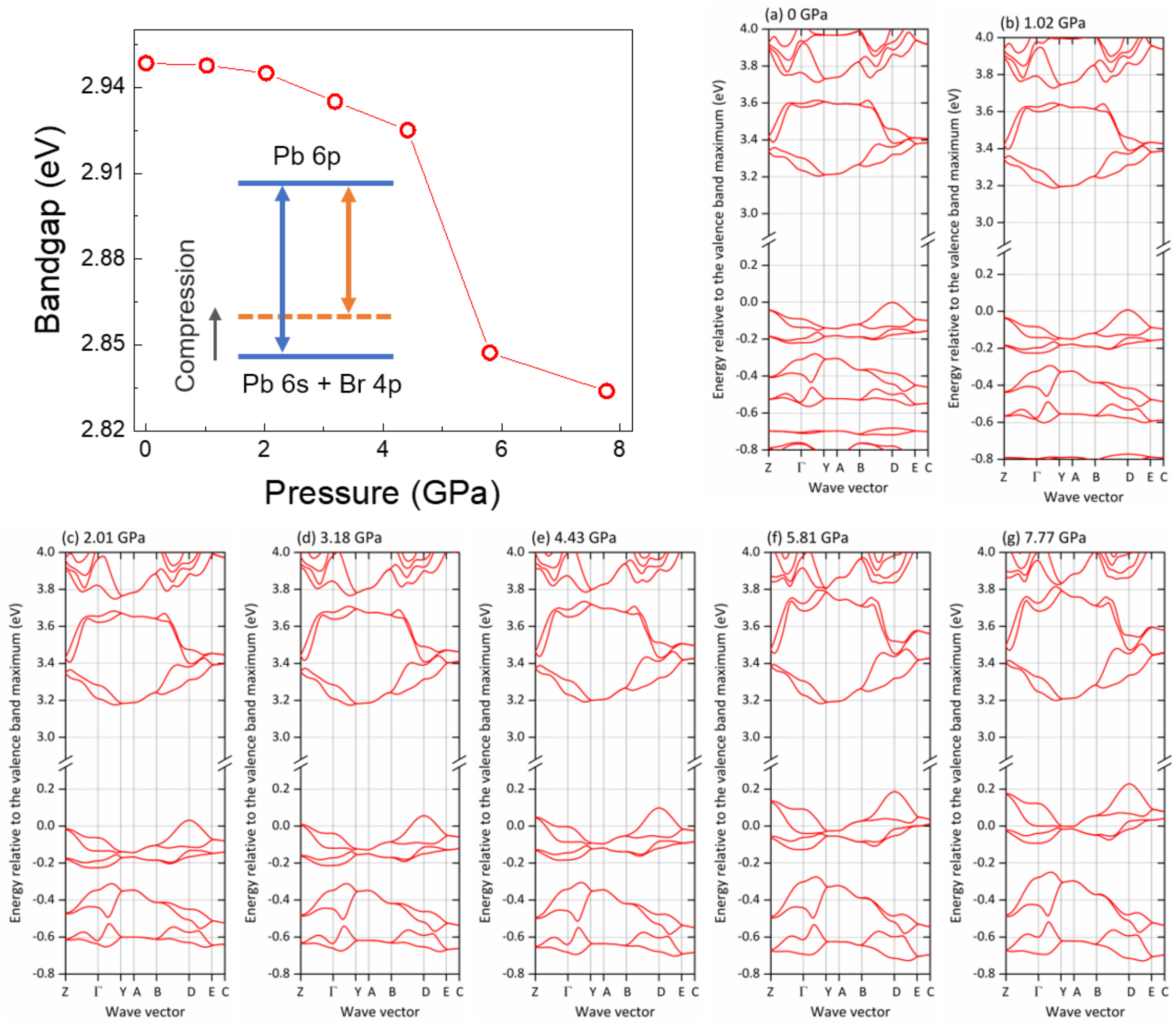

Figure S11. The pressure dependence of bandgap and band structures of $C_5N_2H_{16}Pb_2Br_6$ derived from DFT calculations.

.

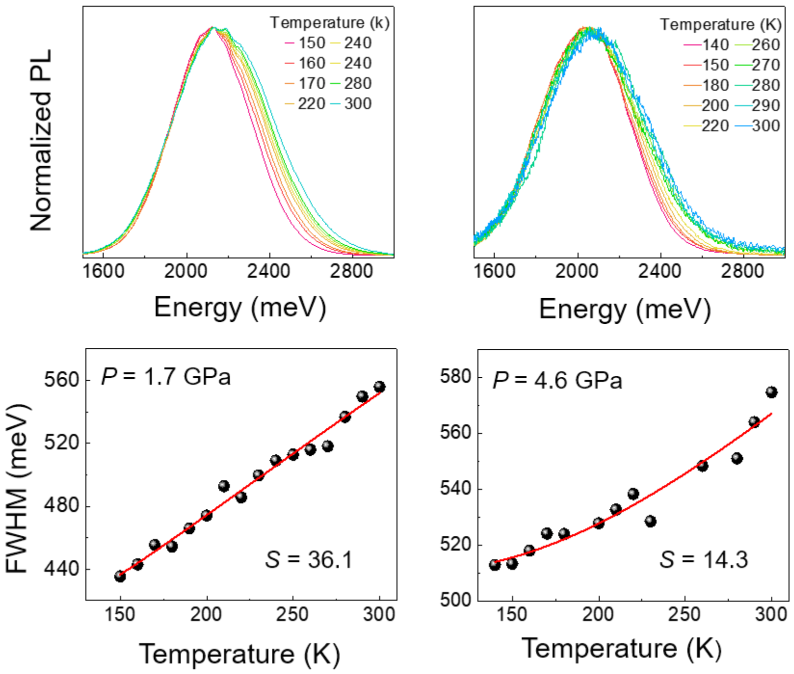

Figure S12. The temperature-dependent photoluminescence spectra (top) and the corresponding fitted values of Huang-Rhys factor S of $C_5H_{16}N_2Pb_2Br_6$ (below) at various pressures of 1.7, 4.6 GPa.

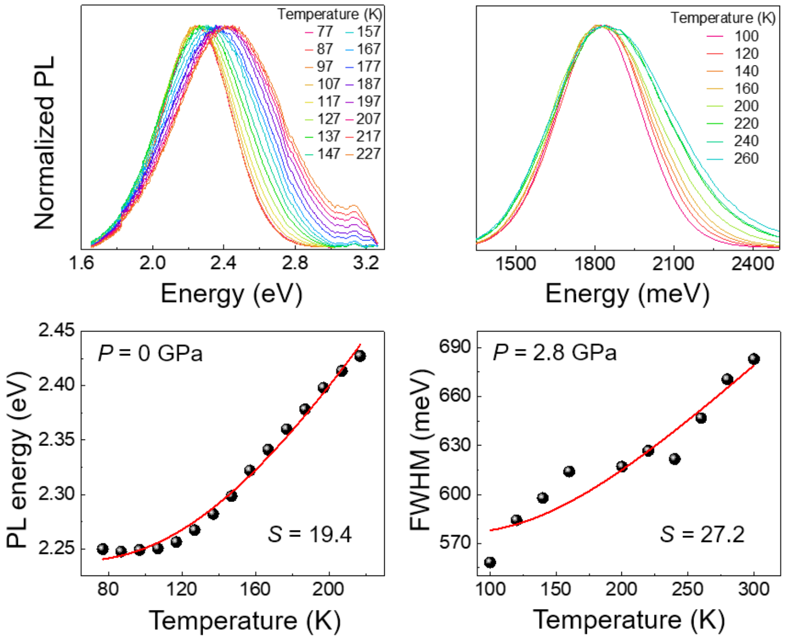

Figure S13. The temperature-dependent photoluminescence spectra (top) and the corresponding fitted Huang-Rhys factor S values of $C_4N_2H_{14}PbBr_4$ and (below) at ambient pressure and 2.8 GPa.

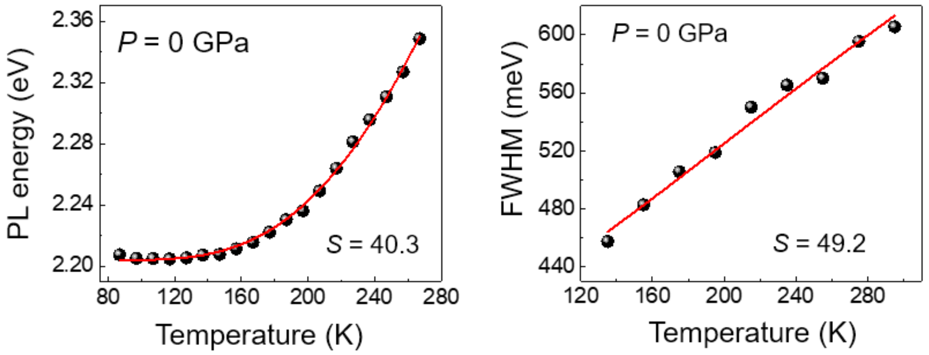

Figure S14. The fitted Huang-Rhys factor *S* values of $C_6N_2H_{16}PbBr_4$ (left) and $(C_6N_2H_{16})_3Pb_2Br_{10}$ (right) at ambient conditions.

Reference

[1] H. K. Mao, J. Xu, P. M. Bell, **1986**, 91, 4673.
[2] C. Prescher, V. B. Prakapenka, *High Pressure Research* **2015**, 35, 223.
[3] a) A. C. Larson, R. B. Von Dreele, *Los Alamos National Laboratory, Los Alamos, NM* **1986**; b) B. H. Toby, E. J. J. o. A. Crystallography, **2001**, 34, 210.
[4] G. Kresse, J. J. P. r. B. Furthmüller, **1996**, 54, 11169.
[5] J. P. Perdew, K. Burke, M. J. P. r. l. Ernzerhof, **1996**, 77, 3865.
[6] a) M. Dion, H. Rydberg, E. Schroder, D. C. Langreth, B. I. Lundqvist, *Phys Rev Lett* **2004**, 92, 246401; b) J. Klimeš, D. R. Bowler, A. J. J. o. P. C. M. Michaelides, **2009**, 22, 022201; c) K. Lee, É. D. Murray, L. Kong, B. I. Lundqvist, D. C. Langreth, *Physical Review B* **2010**, 82.
[7] H. Lin, C. Zhou, J. Neu, Y. Zhou, D. Han, S. Chen, M. Worku, M. Chaaban, S. Lee, E. Berkwits, T. Siegrist, M. H. Du, B. Ma, *Adv. Opt. Mater.* **2019**, 7, 1801474.
[8] a) K. Rurack, M. Spieles, *Analytical chemistry* **2011**, 83, 1232; b) J. R. Lakowicz, *Principles of Fluorescence Spectroscopy*, Springer, **2006**.
[9] a) J. Law, R. Rennie, *A Dictionary of Physics*, Oxford University Press, **2009**; b) G. Kleideiter, M. D. Lechner, W. Knoll, *Macromolecular Chemistry and Physics* **1999**, 200, 1028.
[10] X. Gong, O. Voznyy, A. Jain, W. Liu, R. Sabatini, Z. Piontkowski, G. Walters, G. Bappi, S. Nokhrin, O. Bushuyev, *Nat. Mater.* **2018**, 17, 550.
[11] F. Hong, B. Yue, N. Hirao, G. Ren, B. Chen, H.-K. Mao, *Appl. Phys. Lett.* **2016**, 109.
[12] S. Wang, J. Zhu, Y. Zhang, X. Yu, J. Zhang, W. Wang, L. Bai, J. Qian, L. Yin, N. S. Sullivan, C. Jin, D. He, J. Xu, Y. Zhao, *Proc. Natl. Acad. Sci. USA* **2015**, 112, 15320.
[13] F. Birch, *Phys. Rev.* **1947**, 71, 809.
[14] *Journal of Modern Optics* **1990**, 37, 1688.
[15] a) S. Li, J. Luo, J. Liu, J. Tang, *J. Phys. Chem. Lett.* **2019**, 10, 1999; b) J. Luo, X. Wang, S. Li, J. Liu, Y. Guo, G. Niu, L. Yao, Y. Fu, L. Gao, Q. Dong, C. Zhao, M. Leng, F. Ma, W. Liang, L. Wang, S. Jin, J. Han, L. Zhang, J. Etheridge, J. Wang, Y. Yan, E. H. Sargent, J. Tang, *Nature* **2018**, 563, 541.

[16] a) W. B. Fowler, *Physics of color centers*, Academic Press, New York **1968**; b) K. M. McCall, C. C. Stoumpos, S. S. Kostina, M. G. Kanatzidis, B. W. Wessels, *Chem. Mater.* **2017**, 29, 4129; c) H. Siddique, Z. Xu, X. Li, S. Saeed, W. Liang, X. Wang, C. Gao, R. Dai, Z. Wang, Z. Zhang, *J. Phys. Chem. Lett.* **2020**, 11, 9572; d) W. Stadler, D. M. Hofmann, H. C. Alt, T. Muschik, B. K. Meyer, E. Weigel, G. Muller-Vogt, M. Salk, E. Rupp, K. W. Benz, *Phys. Rev. B Condens Matter* **1995**, 51, 10619.
[17] a) K. P. O'Donnell, X. Chen, *Appl. Phys. Lett.* **1991**, 58, 2924; b) C. Ji, Y. Zhang, T. Zhang, W. Liu, X. Zhang, H. Shen, Y. Wang, W. Gao, Y. Wang, J. Zhao, W. W. Yu, *J. Phys. Chem. C* **2015**, 119, 13841; c) Y. Song, X. Zhang, L. Li, Z. Mo, J. Xu, S. Yu, X. Liu, J. Zhang, *Mater Res Express* **2019**, 6; d) X. \Lao, Z. Yang, Z. Su, Z. Wang, H. Ye, M. Wang, X. Yao, S. Xu, *Nanoscale* **2018**, 10, 9949; e) M. S. Gaponenko, A. A. Lutich, N. A. Tolstik, A. A. Onushchenko, A. M. Malyarevich, E. P. Petrov, K. V. Yumashev, *Phys. Rev. B* **2010**, 82.
[18] a) K. Huang, A. Rhys, in *Selected Papers Of Kun Huang: (With Commentary)*, World Scientific, 2000, 74; b) T. D. Krauss, F. W. J. P. R. B. Wise, *Phys. Rev. B* **1997**, 55, 9860.
